# Cardiac Parameters Better Predict ICU Admission and Short-Term Mortality in Hospitalized Patients With COVID-19

**DOI:** 10.7759/cureus.46141

**Published:** 2023-09-28

**Authors:** Emrah Aksakal, Sidar Ş Aydın, Selim Aydemir, İbrahim Saraç, Faruk Aydınyılmaz, Murat Özmen, Oktay Gülcü, Oğuzhan Birdal, Kamuran Kalkan, Mustafa Öztürk

**Affiliations:** 1 Cardiology, Erzurum City Hospital, Erzurum, TUR; 2 Cardiology, Ataturk University Faculty of Medicine, Erzurum, TUR; 3 Cardiology, Ankara Etlik City Hospital, Ankara, TUR; 4 Cardiology, Ahi Evren Thoracic and Cardiovascular Surgery Training and Research Hospital, Trabzon, TUR

**Keywords:** icu admission, mortality, cardiac parameters, blood parameters, covid-19

## Abstract

Background

COVID-19 is a multisystemic disease that affects many organs, and the use of some parameters is recommended both during hospitalization and follow-up. In this study, we investigated the relationship between blood (liver and kidney function tests, lactate, and D-dimer), infection (C-reactive protein (CRP), lymphocyte count, ferritin, and albumin), and cardiac (creatine kinase-myocardial band (CK-MB), troponin, and brain natriuretic peptide (BNP)) parameters with intensive care unit (ICU) admission and mortality.

Materials and methods

Patients hospitalized in Erzurum City Hospital with the diagnosis of COVID-19 between April 2020 and November 2022 were included in this retrospective study. The patient's files and electronic media records were retrospectively reviewed, and the patient's anamnesis, physical examination, clinical findings, biochemical parameters, and treatment methods were recorded. The ICU needs of the patients and the treatment processes in intensive care were found in the in-hospital records. The hospital records and six-month mortality data were obtained retrospectively with the necessary permissions. Thus, blood parameters and their relation to each other in terms of prognosis were evaluated in determining the six-month mortality rates of the patients and estimating the need for ICU.

Results

A total of 5100 patients were included in the study. The mean age of patients with mortality was 74.2 ± 11.2 and that without mortality was 59.9 ± 15.7 (p < 0.001). In the mortality (+) group, 61.5% of patients were male, and in the mortality (-) group, 47.4% of the patients were male (p < 0.001). The mean age of patients with ICU admission was 69.6 ± 13.6 and without ICU admission was 60.3 ± 15.9 years (p < 0.001). In the ICU admission (+) group, 60.5% of patients were male; and in the ICU admission (-) group, 47.2% of patients were male (p < 0.001). Death and ICU admission were observed more frequently in elderly and male patients (p < 0.001 for both mortality and ICU admission).

Blood parameters were evaluated both in the mortality and ICU groups, and organ function tests, blood count parameters, inflammatory markers, and cardiac parameters were significantly associated with poor outcomes. Cox regression analysis showed that lactate, albumin, Ln(troponin), and Ln(BNP) were independent predictors of mortality and ICU admission. Receiver operating characteristics (ROC) curve analysis showed that Ln(troponin) and Ln(BNP) levels predicted the development of mortality and ICU admission better than other parameters.

Discussion

COVID-19 can cause problems in different systems as a result of an inflammatory response, secreted cytokines, hypercoagulability, and direct tissue damage. When treating patients, a more appropriate approach is to evaluate different parameters together rather than focusing on a single parameter and deciding accordingly. However, evaluating alterations in many parameters in a disease that affects many systems is difficult and increases the risk of mistakes. Although each blood parameter separately is important, it was observed that the cardiac parameters troponin I and BNP have better predictive values than others in predicting the course and prognosis of COVID-19.

Conclusion

Blood parameters are used in COVID-19 diagnosis, treatment, and follow-up. Although it is not primarily a cardiac disease, cardiac markers can provide better results in showing the course and prognosis of COVID-19.

## Introduction

SARS-CoV-2 is the causative agent of acute COVID-19 respiratory disease and belongs to the Coronaviridae family. Although the disease started in 2019, the viral variants still spread around the world. SARS-CoV-2, which caused the COVID-19 pandemic, affects many systems in the body. It is a common cause of mortality and morbidity owing to multi-organ involvement [1,2].

Owing to the uncertainty of the disease, many countries have developed different diagnosis and treatment methods. In the guide of the Ministry of Health of the Republic of Turkey, algorithms are given on how to follow the diagnosis and treatment of patients with COVID-19 [3,4]. Important information, such as which patients should be hospitalized and which patients need intensive care, is also given in this guide.

Because COVID-19 is a multisystemic disease that affects many organs, the use of some parameters both during hospitalization and follow-up is recommended. Coagulation and perfusion parameters are used to indicate damage to the vascular bed, inflammation parameters are used because the virus causes widespread inflammation in the body, and organ-specific parameters are used because of the acute toxic effect that damages the organs. Therefore, in the current guideline, the use of kidney and liver function tests (creatinine, aspartate aminotransferase (AST) and alanine aminotransferase (ALT)), blood count parameters (lymphocytes), infection parameters (ferritin and C-reactive protein (CRP)), lactate, D-dimer, and troponin parameters is recommended [4].

In this study, we investigated the relationship between blood parameters (liver and kidney function tests, lactate, and D-dimer), infection parameters (CRP, lymphocyte count, ferritin, and albumin), and cardiac parameters (creatine kinase-myocardial band (CK-MB), troponin, and brain natriuretic peptide (BNP)) with intensive care unit (ICU) admission and six-month mortality.

## Materials and methods

Patients hospitalized in Erzurum City Hospital with the diagnosis of COVID-19 between April 2020 and November 2022 were included in this retrospective study. The patient's files and electronic media records were retrospectively reviewed, and the patient's anamnesis, physical examination, clinical findings, biochemical parameters, and treatment methods were recorded. The ICU needs of the patients and the treatment processes in intensive care were found in in-hospital records. The hospital records and six-month mortality data were obtained retrospectively with the necessary permissions. Thus, blood parameters taken during hospitalization and their relation to each other in terms of prognosis were evaluated in determining the six-month mortality rates of the patients and estimating the need for ICU.

COVID-19 definition

Possible cases admitted to Erzurum City Hospital between April 2020 and November 2022 were evaluated according to the guidelines of the Ministry of Health of the Republic of Turkey. Oral and nasal swab samples were taken from patients with suspected COVID-19, and polymerase chain reaction (PCR) was used for molecular analysis. Pulmonary computerized tomography (CT) was applied to selected patients deemed appropriate by the examining clinician [3]. Complete blood count (CBC), CRP, and biochemistry tests are routinely performed on patients who attend the emergency department with complaints compatible with COVID-19. CBC was performed using the Sysmex XN-3100 Automated Hematology System (Sysmex, TOA Medical Electronics, Kobe, Japan, and distributed by American Scientific Products, Chicago, IL). D-dimer was measured by particle-enhanced immunoturbidimetric assays (InnovanceDdimer) on a Siemens BCSXP Systems automated coagulation analyzer (Siemens Healthcare GmbH, Marburg, Germany). A second swab sample was taken from hospitalized patients when the first sample was negative. When one of the two samples taken was positive, the patient was diagnosed with COVID-19, and if both were negative, and the CT was negative, COVID-19 was excluded.

ICU admission criteria

In the cases specified in the guideline, the patients were evaluated by the relevant clinician, and the need for intensive care was decided [4].

-Patient with dyspnea and respiratory distress despite oxygen therapy and in the follow-up; oxygen requirement increased.

-Respiration rate ≥ 30/min.

-PaO_2_/FiO_2_ < 300.

-SpO_2_ < 90% or PaO_2_ < 70 mmHg despite 5 L/min oxygen therapy.

-Hypotension (systolic blood pressure < 90 mmHg and more than 40 mmHg); decrease from normal systolic blood pressure and mean arterial pressure < 65 mmHg; tachycardia > 100/min.

-Patients with acute kidney injury, acute liver function tests, confusion, acute organ dysfunction such as acute bleeding diathesis and immunosuppression.

-Troponin elevation and arrhythmia.

-Lactate > 2 mmol.

-Presence of skin disorders such as capillary return disorder and cutis marmorata.

Statistical analysis

Data were analyzed using the SPSS 23.0 version (IBM, Armonk, NY, USA). Continuous variables were expressed as mean ± standard deviation or median (interquartile range), and categorical variables were expressed as percentages. Whether continuous variables fit the normal distribution was determined using the “homogeneity of variance” test. Continuous variables were compared using the "student’s t-test” and the “Mann-Whitney U test” as appropriate. Categorical variables were compared using the “chi-square” test. To find independent predictors of mortality and ICU admission, the Cox regression model was used, and parameters that were significant in univariate analysis were included in the model. While determining the parameters to be included in the regression analysis, the correlation coefficients were calculated. Parameters with high and very high correlations were not included in the regression analysis. The receiver operating characteristics (ROC) curve analysis was performed to determine the sensitivity and specificity values of parameters in the prediction of mortality and ICU admission. Negatively correlated parameters were included in the analysis as n-1 to avoid visual misinterpretation. Variables were considered statistically significant when the P-value was < 0.05.

## Results

A total of 5100 patients were included in the study. The mean age of patients with mortality was 74.2 ± 11.2 and that without mortality was 59.9 ± 15.7 (p < 0.001). In the mortality (+) group, 61.5% of patients were male; and in the mortality (-) group, 47.4% of the patients were male (p < 0.001). The mean age of patients with ICU admission was 69.6 ± 13.6 and without ICU admission was 60.3 ± 15.9 years (p < 0.001). In the ICU admission (+) group, 60.5% of patients were male; and in the ICU admission (-) group, 47.2% of patients were male (p < 0.001).

Death and ICU admission were observed more frequently in elderly and male patients (p < 0.001 for both mortality and ICU admission). In addition, comorbid diseases such as hypertension (HT), diabetes mellitus (DM), coronary artery disease (CAD), and chronic obstructive pulmonary disease (COPD) were significantly more frequent in patients with the mortality (+) group and ICU admission (+) group. Although no significant difference was observed in the PCR tests of these patients, pulmonary involvement was observed more frequently in both the mortality and ICU groups (p < 0.001 for both). Baseline demographic data are shown in Table 1.

Blood parameters were evaluated in the mortality and ICU groups, and the results showed that organ function tests, blood count parameters, inflammatory markers, and cardiac parameters in both groups were significantly associated with poor outcomes. The comparison of blood parameters is shown in Table 1.

**Table 1:** Comparison of baseline demographic data of the cohort by mortality and intensive care unit admission. HT: hypertension; DM: diabetes mellitus; CAD: coronary artery disease; COPD: chronic obstructive pulmonary disease; PCR: polymerase chain reaction; CT: computerized tomography; ALT: alanine aminotransferase; Hgb: hemoglobin; WBC: white blood cell; CRP: C-reactive protein; CK-MB: creatine kinase-myocardial band; BNP: brain natriuretic peptide.

Variables	Mortality + (n=667)	Mortality – (n=4433)	P	ICU + (n=795)	ICU – (n=4305)	P
Age	74.2 ± 11.2	59.9 ± 15.7	<0.001	69.6 ± 13.6	60.3 ± 15.9	<0.001
Gender (male, %)	410 (61.5)	2101 (47.4)	<0.001	481 (60.5)	2030 (47.2)	<0.001
HT (n, %)	443 (66.4)	2118 (47.8)	<0.001	488 (61.4)	2073 (48.2)	<0.001
DM (n, %)	204 (30.6)	1205 (27.2)	0.069	243 (30.6)	1166 (27.1)	0.046
CAD (n, %)	210 (31.5)	892 (20.1)	<0.001	244 (30.7)	858 (19.9)	<0.001
COPD (n, %)	153 (22.9)	213 (11.6)	<0.001	147 (18.5)	519 (12.1)	<0.001
Positive PCR (n, %)	495 (74.2)	3202 (72.2)	0.285	590 (74.2)	3107 (72.2)	0.236
Positive CT (n, %)	655 (98.2)	3998 (90.2)	<0.001	771 (97)	3882 (90.2)	<0.001
Glucose (mg/dL)	183 ± 71	151 ± 68	<0.001	176 ± 65	151 ± 69	<0.001
Creatinine (mg/dL)	1.29 (0.96-1.99)	0.85 (072-1.05)	<0.001	1.14 (0.83-1.77)	0.86 (0.72-1.06)	<0.001
ALT (U/L)	36 (22-72)	31.5 (22-48)	<0.001	40 (25-73)	31 (21-47)	<0.001
Lactate (mmol/L)	3.24 ± 1.87	2.16 ± 0.9	<0.001	3.1 ± 1.58	2.2 ± 1	<0.001
D-dimer (μg/mL)	2488 (759-7500)	288 (90-910)	<0.001	2311 (712-7287)	269 (88-830)	<0.001
Hgb (g/dL)	12.5 ± 2.6	13.3 ± 1.7	<0.001	12.5 ± 2.5	13.4 ± 1.7	<0.001
WBC (10^3^/µL)	10.57 (7.63-13.85)	6.85 (5.32-8.85)	<0.001	10 (7.44-13.17)	6.81 (5.3-8.78)	<0.001
Lymphocyte (10^3^/µL)	0.72 (0.49-1.03)	1.3 (0.95-1.73)	<0.001	0.76 (0.53-1.14)	1.3 (0.96-1.73)	<0.001
Neutrophile (10^3^/µL)	9.03 (6.22-11.82)	4.75 (3.41-6.74)	<0.001	8.52 (5.83-11.37)	4.73 (3.38-6.68)	<0.001
Albumin (g/L)	32 ± 4.7	38 ± 4.6	<0.001	32 ± 4.7	38 ± 4.6	<0.001
Ferritin (ng/mL)	632 (309-1169)	236 (103-476)	<0.001	613 (281-1138)	232 (102-458)	<0.001
CRP (mg/L)	97.2 (57-147)	31 (11-62)	<0.001	84 (46-140)	31 (11-62)	<0.001
Procalcitonin (ng/mL)	1.1 (0.36-3.32)	0.08 (0.02-0.34)	<0.001	0.86 (0.25-2.74)	0.08 (0.02-033)	<0.001
CK-MB (U/L)	30 (20-60)	20 (10-25)	<0.001	30 (20-50)	20 (10-23)	<0.001
Troponin I (ng/mL)	135.1 (6.6-1080)	2.5 (0.1-9.5)	<0.001	88.4 (6.7-931.2)	2.5 (0.1-9.1)	<0.001
BNP (pg/mL)	7074 (1529-21326)	145 (43-609)	<0.001	4923 (978-18662)	134 (42-471)	<0.001

The Cox regression model was applied to identify independent predictors of mortality development and parameters that were significant in univariate analysis were included in the regression model. Of these parameters, lactate, albumin, Ln(troponin), and Ln(BNP) levels were independent predictors of mortality (p = 0.003 for lactate, p = 0.002 for albumin, p = 0.002 for Ln(troponin), and p < 0.001 for Ln(BNP)). The regression analysis results for mortality are shown in Table 2.

**Table 2:** Regression analysis of blood, inflammation, and cardiac parameters in predicting mortality. ALT: alanine aminotransferase; CRP: C-reactive protein; CK-MB: creatine kinase-myocardial band; BNP: brain natriuretic peptide.

Variables	Univariate OR, 95% CI	P	Multivariate OR, 95% CI	P
Creatinine	1.42 (1.37-1.47)	<0.001	1 (0.98-1.01)	0.587
ALT	1.02 (1.02-1.03)	<0.001	1 (0.99-1.01)	0.413
Lactate	1.38 (1.34-1.41)	<0.001	1.18 (1.06-1.32)	0.003
Ln(D-dimer)	3.08 (2.84-3.34)	<0.001	1.1 (0.85-1.41)	0.464
Lymphocyte	1.03 (1.01-1.06)	<0.001	1.01 (0.98-1.03)	0.511
Albumin	1.16 (1.14-1.18)	<0.001	0.55 (0.38-0.80)	0.002
Ferritin	1.02 (1.01-1.03)	<0.001	1 (0.99-1.01)	0.558
CRP	1.03 (1.02-1.04)	<0.001	1 (0.98-1.02)	0.715
Procalcitonin	1.04 (1.03-1.05)	<0.001	1.02 (0.98-1.04)	0.276
CK-MB	1.03 (1.02-1.04)	<0.001	1 (0.98-1.02)	0.971
Ln(Troponin)	2.15 (2.04-2.28)	<0.001	1.29 (1.1-1.52)	0.002
Ln(BNP)	3.84 (3.23-4.55)	<0.001	1.93 (1.47-2.53)	<0.001

**Figure 1:**
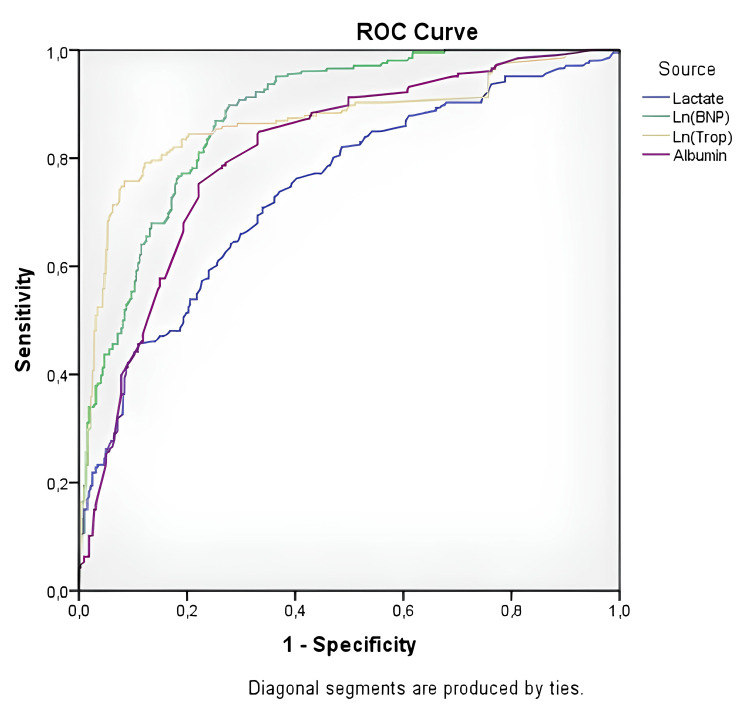
ROC curve analysis of the significantly altered parameters in the mortality regression analysis (1/albumin was used because albumin is a negative acute phase reactant). ROC: receiver operating characteristics, BNP: brain natriuretic peptide.

ROC curve analysis was performed to identify significantly altered parameters in the mortality estimation. The results showed that Ln(troponin) and Ln(BNP) levels better predicted mortality (Ln(troponin) sensitivity: 82%, specificity: 81.9%, area under the curve (AUC): 0.867 (0.832-0.902), p < 0.001; Ln(BNP) sensitivity: 78.2%, specificity: 78.2%, AUC: 0.879 (0.851-0.907), p < 0.001). The ROC curve analysis results for mortality are shown in Figure 1.

The Cox regression model was applied to identify the independent determinants of ICU admission in the parameters found in univariate analysis. Similar to the mortality group, lactate, albumin, Ln(troponin), and Ln(BNP) levels were independent predictors of ICU hospitalization (p = 0.002 for lactate, p < 0.001 for albumin, p < 0.001 for Ln(troponin), and p < 0.001 for Ln) (BNP)). The results of the regression analysis for ICU admission are shown in Table 3.

**Table 3:** Regression analysis of blood, inflammation, and cardiac parameters in predicting ICU admission. ALT: alanine aminotransferase; CRP: C-reactive protein; CK-MB: creatine kinase-myocardial band; BNP: brain natriuretic peptide.

Variables	Univariate OR, 95% CI	P	Multivariate OR, 95% CI	P
Creatinine	1.39 (1.34-1.44)	<0.001	1.02 (0.93-1.12)	0.673
ALT	1.04 (1.03-1.04)	<0.001	1 (0.99-1.01)	0.478
Lactate	1.38 (1.34-1.42)	<0.001	1.19 (1.06-1.31)	0.002
Ln(D-dimer)	3.15 (2.92-3.39)	<0.001	1.14 (0.92-1.43)	0.235
Lymphocyte	1.03 (1.01-1.06)	<0.001	1.01 (0.98-1.03)	0.505
Albumin	1.16 (1.15-1.18)	<0.001	0.51 (0.36-0.72)	<0.001
Ferritin	1.02 (1.01-1.03)	<0.001	1 (0.98-1.02)	0.150
CRP	1.03 (1.02-1.03)	<0.001	1 (0.99-1.01)	0.936
Procalcitonin	1.03 (1.02-1.03)	<0.001	1.01 (0.98-1.03)	0.325
CK-MB	1.02 (1.01-1.03)	<0.001	1 (1-1.01)	0.056
Ln(Troponin)	2.31 (2.19-2.43)	<0.001	1.38 (1.18-1.60)	<0.001
Ln(BNP)	3.42 (2.95-3.96)	<0.001	1.56 (1.24-1.98)	<0.001

**Figure 2:**
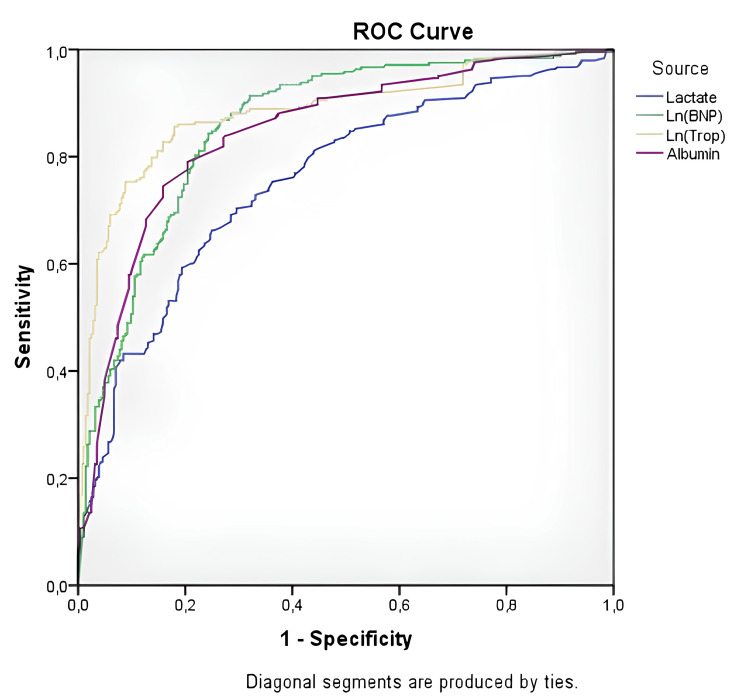
ROC curve analysis of the significantly altered parameters in the ICU admission regression analysis (1/albumin was used because albumin is a negative acute phase reactant). ROC: receiver operating characteristics, BNP: brain natriuretic peptide.

ROC curve analysis was performed to identify significantly altered parameters in the estimation of ICU admission. Similar to the mortality group, Ln(troponin) and Ln(BNP) levels better predicted intensive care admission (Ln(troponin) sensitivity: 82.7%, specificity: 82.7%, AUC: 0.884 (0.853-0.914), p < 0.001; Ln(BNP) sensitivity: 78.6%, specificity: 78.5%, AUC: 0.858 (0.826-0.890), p < 0.001). The ROC curve analysis results for ICU admission are shown in Figure 2 and Table 4.

**Table 4:** ROC curve analysis results of parameters in predicting death and ICU admission. AUC: area under the curve; SE: standard error; ICU: intensive care unit; BNP: brain natriuretic peptide, ROC: receiver operating characteristics.

Variables	Sensitivity (%)	Specificity (%)	AUC (95% CI)	SE	P
Six-month mortality					
Lactate	68	67	0.741 (0.697-0.785)	0.022	<0.001
Albumin	75.2	77.9	0.810 (0.772-0.847)	0.019	<0.001
Ln(Troponin)	82	81.9	0.867 (0.832-0.902)	0.018	<0.001
Ln(BNP)	78.2	78.2	0.879 (0.851-0.907)	0.014	<0.001
ICU admission					
Lactate	70.4	70.1	0.757 (0.715-0.798)	0.021	<0.001
Albumin	79	79.6	0.845 (0.811-0.879)	0.017	<0.001
Ln(Troponin)	82.7	82.7	0.884 (0.853-0.914)	0.016	<0.001
Ln(BNP)	78.6	78.5	0.858 (0.826-0.890)	0.016	<0.001

## Discussion

In this large study population analysis, we found that many parameters can be used to predict the course and short-term prognosis of COVID-19, but cardiac parameters had a better predictive value than other parameters.

COVID-19 is a contagious and deadly disease that affects many systems. It remains important owing to the problems experienced during the pandemic and the morbidity and mortality caused by the disease. As a result of the mutations and changes it has undergone, the virus remains a threat to humanity that must be considered.

COVID-19 can cause problems in different systems resulting from the inflammatory response, secreted cytokines, hypercoagulability, and direct tissue damage [5-7]. The situation becomes more complex because of the problems caused by the disease and the side effects of the drugs used. Both the course of the disease and the effects of the medications given during treatment must be monitored. Therefore, various parameters are used to evaluate different systems. When evaluating patients, a more appropriate approach is to evaluate different parameters together rather than looking at a single parameter and deciding accordingly.

Various algorithms have been developed for patients who need to be admitted to the ICU based on the progression of the disease, during hospitalization and treatment, and in patients with COVID-19. The COVID-19 guideline of the Ministry of Health of the Republic of Turkey provides recommendations for which patients are suitable for home treatment or need to be hospitalized [3]. Similarly, there are recommendations for the evaluation of the patient in terms of the need for ICU during the first hospitalization or during treatment [4]. However, the need to evaluate many parameters in a disease that affects many systems increases the risk of making mistakes. For this reason, artificial intelligence-based studies have been performed with the aim of predicting the course of the disease or the prognosis [8]. However, these recently developed procedures have not replaced traditional evaluations.

Hemogram and infection parameters are the most frequently used in the diagnosis, course of the disease, and treatment guide for COVID-19. In particular, the decrease in lymphocyte count and albumin (a negative acute phase reactant) levels and the increase in ferritin and CRP (a positive acute phase reactant) levels are related to the severity of the disease [9-12]. We obtained similar results in our study. In fact, among these parameters, albumin was an independent predictor of both ICU admission and six-month mortality.

Liver and kidney function tests are used everywhere in routine practice to assess organ function. They have also been used in COVID-19 both as an indicator of damage and to observe possible side effects of drugs and dehydration; the deterioration of levels is correlated with the severity of the disease [13-15]. These parameters were significantly associated with ICU admission and six-month mortality in our study, but they were not independent predictors.

In COVID-19, a disease with hypercoagulability, lactate levels are used to assess tissue perfusion and D-dimer levels have been frequently monitored to assess the thrombus burden; both parameters are associated with poor outcomes [5,16,17]. In our study, both parameters were associated with ICU admission, and lactate levels were an independent predictor of both ICU admission and six-month mortality.

COVID-19 can lead to cardiac damage and acute coronary syndromes, both by direct tissue damage and the inflammatory response and thrombosis it causes. Cardiac involvement is associated with poor outcomes in almost every disease. In terms of cardiac involvement, troponin I and BNP levels are associated with a poor prognosis [18-20]. Similarly, in our study, these two parameters were associated with ICU admission and six-month mortality. In addition, both parameters were independent predictors of ICU admission and six-month mortality.

Although many parameters examined in our study were associated with ICU admission and six-month mortality, lactate, albumin, troponin, and BNP levels were independent predictors of six-month mortality in the regression analysis performed after the exclusion of related parameters by correlation analysis. In the ROC analysis, although the values were similar, BNP had the best predictive value for six-month mortality; in ICU admission, troponin I had the best predictive value.

Limitations

The main limitations of the study are its retrospective design and being a single-center study. Therefore, the results of the study cannot be generalized. In addition, owing to a lack of information about the disease, a clear diagnosis and treatment protocol could not be established and the treatments applied varied.

## Conclusions

Blood parameters are easy to study and provide much information about diseases being useful in diagnosis, treatment, and prognosis. Similarly, these parameters are used all over the world for COVID-19, which appeared suddenly and with little relevant knowledge.

Although each blood parameter separately is important, it was observed that the cardiac parameters troponin I and BNP had better predictive values than other parameters regarding the course and prognosis of COVID-19.
